# Editorial: Recent advances in childhood vascular tumors

**DOI:** 10.3389/fonc.2024.1465155

**Published:** 2024-07-25

**Authors:** Tong Qiu, Kaiying Yang, Yi Ji

**Affiliations:** ^1^ Department of Pediatric Surgery, West China Hospital, Sichuan University, Chengdu, China; ^2^ Department of Pediatric Surgery, Guangzhou Women and Children’s Medical Center, National Children’s Medical Center for South Central Region, Guangzhou Medical University, Guangzhou, China

**Keywords:** vascular tumors, infantile hemangioma, infantile hepatic hemangioma, Kaposiform hemangioendothelioma, propranolol

Vascular tumors are a type of disease with high incidence, diversity, and complexity during children’s growth and development. Infantile hemangioma (IH) is the most common vascular tumor in children, with an incidence rate of approximately 4%-5%. Although propranolol is effective as a first-line treatment for IH, issues such as drug resistance, rebound, and recurrence frequently occur during treatment. This is especially challenging in the case of high-risk IHs, such as infantile hepatic hemangioma (IHH) (1). Kaposiform hemangioendothelioma (KHE) is a rare and aggressive vascular tumor, and treating KHE patients with Kasabach-Merritt phenomenon (KMP) remains a significant clinical challenge. Therefore, individualized treatment for high-risk vascular tumors and in-depth mechanistic research to explore more effective clinical treatment strategies are of paramount importance. (**Figure 1**) This Research Topic focuses on both clinical and basic research related to vascular tumors, aiming to advance scientific exploration and clinical applications in this field. Four articles were accepted and included in this Research Topic, two of which were published as original research articles, one was a case report and one was a mini review, all of which covered the above topics.

**Figure 1:**
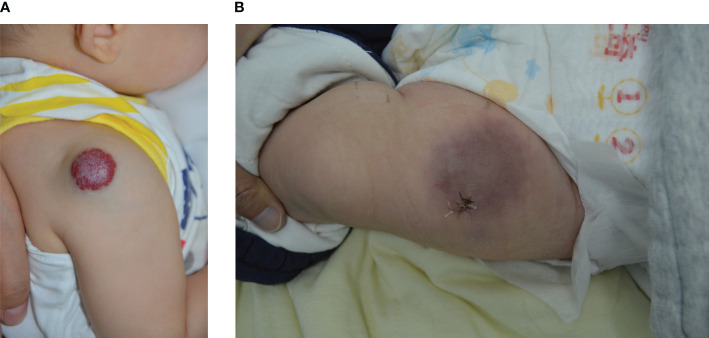
**(A)** Male, 6 months old, with a mixed IH on the right shoulder; **(B)** Female, 5 months old, with KHE on the anterior aspect of the left thigh.

Mast cells (MCs) are widely distributed in various tissues, such as skin and mucous membranes. They participate in multiple physiological and pathological processes, including inflammatory responses and tissue remodeling, by releasing various bioactive substances, such as histamine, cytokines, and growth factors. Increasing research attention has been given to the role of MCs in IH, revealing that MCs may play a crucial role in tumor formation and progression (2, 3). Xia et al. demonstrated that MCs in IH primarily function through two pathways: promoting angiogenesis and regulating immune responses. First, MCs release angiogenic factors such as vascular endothelial growth factor (VEGF) and basic fibroblast growth factor (bFGF) to promote the formation of new blood vessels. Second, inflammatory mediators released by MCs, such as tumor necrosis factor-α (TNF-α) and interleukin-6 (IL-6), can modulate the local immune microenvironment, further supporting tumor growth and expansion. This research offers new insights into the pathological mechanisms of IH. Future studies could further elucidate the specific functions of MCs at various stages of IH, including their roles in tumor proliferation and regression. Additionally, investigating the interactions between MCs and other cell types, such as endothelial cells, fibroblasts, and immune cells, could reveal their combined impact on angiogenesis and the tumor microenvironment. These findings could provide potential applications for targeted IH therapies.

The screening and treatment systems for diffuse infantile hepatic hemangioma (DIHH), a high-risk type of infantile hemangioma, have evolved over the past few decades. This disease can lead to severe complications such as hypothyroidism, consumptive coagulopathy, and high-output congestive heart failure, posing life-threatening risks to patients (4). Particularly for patients with more than five cutaneous IHs, IHH screening is recommended (5). Currently, oral propranolol is becoming an effective systemic treatment for IHH, resulting in significant tumor reduction and symptom relief. Li et al. described a case of an infant with DIHH who showed remarkable improvement after receiving oral propranolol treatment without severe side effects. This case further confirms the potential of propranolol for treating DIHH. A retrospective 30-year study on IHH revealed that among 124 patients, 6% had DIHH. In the era of propranolol use, the proportion of IHH patients requiring surgery significantly decreased [pre-propranolol era 25/48 (52%) vs post-propranolol era 16/76 (21%); P = 0.0003] (6). Some IHH patients are asymptomatic and can be observed until spontaneous resolution, but most patients require complex multidisciplinary treatment approaches. Propranolol did not eliminate the need for surgery. Another study on the monitoring and treatment of IHH suggested that staged pharmacotherapy should be used for treating IHH, but more aggressive surgical intervention for tumors unresponsive to initial pharmacotherapy may help shorten treatment duration and improve outcomes (7).

Propranolol has achieved significant success as a first-line treatment for IH. However, whether the combination of the sclerosing agent lauromacrogol with propranolol can further enhance treatment efficacy and safety is currently a research hotspot. Ma et al. conducted a study comparing the efficacy and safety of oral propranolol alone versus in combination with intralesional injections of lauromacrogol for treating KHE. The patients were divided into two groups: one group received only oral propranolol, and the other group received oral propranolol combined with lauromacrogol. Although the combination treatment group showed an advantage in terms of the secondary response rate, there was no significant improvement in the final cure rate or reduction in treatment duration. Future research could explore the optimal application scheme of lauromacrogol at different doses and frequencies and investigate the combined mechanism of propranolol and lauromacrogol to develop more effective personalized treatment strategies. In addition, the potential side effect of lauromacrogol injection in young children deserves further study.

The diagnosis and treatment of KHE associated with KMP often pose significant challenges. Li et al. conducted a comprehensive review of treatment experiences for different risk groups for KHE, providing valuable insights into managing this complex disease. The study classified patients into three clinicopathological stages based on tumor invasion depth and categorized KHE into three risk levels according to the severity of thrombocytopenia: low-risk, high-risk, and extremely high-risk groups. Treatment strategies varied across these risk groups. For low-risk patients, surgical treatment was the primary choice, and no patients who underwent surgery experienced recurrence during the maximum follow-up of five years. In the high-risk group, 92% of patients received surgical treatment, whereas the treatment for extremely high-risk patients was more complex. Sirolimus has demonstrated significant advantages in treating KHE (8). Whether used alone for KHE without KMP or in combination with steroids for KHE with KMP, more than 90% of patients experienced substantial symptom relief and/or improvement in complications. In a large multicenter cohort study of 159 patients, the incidence of KMP was consistent with that in previous research, but the mortality rate (1.3%) was much lower (9). The overall treatment response rate was high (>70%), highlighting the critical role of sirolimus in KHE management.

## Conclusion

This research explored and discussed several key problems in the current pathogenesis and treatment of vascular tumors through the above four articles. We should not only explore the mechanism underlying the successful treatment of IHs by propranolol but also address the challenges associated with refractory vascular tumors. These recent developments and efforts are significant for the future management of vascular tumors.

## Author contributions

TQ: Conceptualization, Data curation, Investigation, Writing – original draft, Writing – review & editing. KY: Conceptualization, Data curation, Investigation, Writing – original draft, Writing – review & editing. YJ: Conceptualization, Funding acquisition, Investigation, Writing – original draft, Writing – review & editing.
